# A Protein Microarray for the Rapid Screening of Patients Suspected of Infection with Various Food-Borne Helminthiases

**DOI:** 10.1371/journal.pntd.0001899

**Published:** 2012-11-29

**Authors:** Jia-Xu Chen, Mu-Xin Chen, Lin Ai, Jun-Hu Chen, Shao-Hong Chen, Yong-Nian Zhang, Yu-Chun Cai, Xing-Quan Zhu, Xiao-Nong Zhou

**Affiliations:** 1 National Institute of Parasitic Diseases, Chinese Center for Disease Control and Prevention, WHO Collaborating Center for Malaria, Schistosomiasis and Filariasis, Key Laboratory of Parasite and Vector Biology, Ministry of Health, Shanghai, People's Republic of China; 2 State Key Laboratory of Veterinary Etiological Biology, Key Laboratory of Veterinary Parasitology of Gansu Province, Lanzhou Veterinary Research Institute, CAAS, Lanzhou, People's Republic of China; Centers for Disease Control and Prevention, United States of America

## Abstract

**Background:**

Food-borne helminthiases (FBHs) have become increasingly important due to frequent occurrence and worldwide distribution. There is increasing demand for developing more sensitive, high-throughput techniques for the simultaneous detection of multiple parasitic diseases due to limitations in differential clinical diagnosis of FBHs with similar symptoms. These infections are difficult to diagnose correctly by conventional diagnostic approaches including serological approaches.

**Methodology/Principal Findings:**

In this study, antigens obtained from 5 parasite species, namely *Cysticercus cellulosae*, *Angiostrongylus cantonensis*, *Paragonimus westermani*, *Trichinella spiralis* and *Spirometra* sp., were semi-purified after immunoblotting. Sera from 365 human cases of helminthiasis and 80 healthy individuals were assayed with semi-purified antigens by both a protein microarray and the enzyme-linked immunosorbent assay (ELISA). The sensitivity, specificity and simplicity of each test for the end-user were evaluated. The specificity of the tests ranged from 97.0% (95% confidence interval (CI): 95.3–98.7%) to 100.0% (95% CI: 100.0%) in the protein microarray and from 97.7% (95% CI: 96.2–99.2%) to 100.0% (95% CI: 100.0%) in ELISA. The sensitivity varied from 85.7% (95% CI: 75.1–96.3%) to 92.1% (95% CI: 83.5–100.0%) in the protein microarray, while the corresponding values for ELISA were 82.0% (95% CI: 71.4–92.6%) to 92.1% (95% CI: 83.5–100.0%). Furthermore, the Youden index spanned from 0.83 to 0.92 in the protein microarray and from 0.80 to 0.92 in ELISA. For each parasite, the Youden index from the protein microarray was often slightly higher than the one from ELISA even though the same antigen was used.

**Conclusions/Significance:**

The protein microarray platform is a convenient, versatile, high-throughput method that can easily be adapted to massive FBH screening.

## Author Summary

Food-borne helminthiases (FBHs) have caused significant problems in public health and also posed socio-economic concerns. Common FBHs, such as cysticercosis, trichinellosis, paragonimiasis, sparganosis and angiostrongyliasis, have a worldwide distribution with high morbidity and even death. The objective of the present study was to develop and test a rapid assay suitable for large-scale screening for FBHs that would also allow differential diagnosis between the various parasite species. We tested archived, well-characterized serum specimens and prioritized tests for future evaluation in rapid and simultaneous screening of five different FBHs, i.e. cysticercosis, trichinellosis, paragonimiasis, sparganosis and angiostrongyliasis. This was done with a multiplex protein microarray assay equipped with semi-purified antigens capable of detecting disease-specific antibodies. Results showed that the protein microarray developed displayed a good specificity, ranging from 97.0% to 100.0%, and a sensitivity, ranging from 85.7% to 92.1%, with a Youden index variation from 0.83 to 0.92. It was concluded that the protein microarray provides a sensitive, high-throughput technique for the simultaneous detection of multiple FBHs overcoming the limitations of conventional diagnostics.

## Introduction

Food-borne helminthiases (FBHs) are considered neglected tropical diseases (NTDs) by the World Health Organization (WHO). These infections are increasingly found to cause public health problems and pose socio-economic concerns worldwide. FBHs are caused by infections by helminths through consumption of infected or contaminated water and food [1]–[8]. The five most common FBHs, namely cysticercosis, trichinellosis, paragonimiasis, sparganosis and angiostrongyliasis, are not only the most important FBHs in the People's Republic of China (P.R. China) but have also a worldwide distribution. Multiple infections are common and co-endemicity areas have been found with more than two different FBHs overlapping geographically.

Numerous reports describing the adverse impact of FBHs on human health have appeared, some of which reporting particularly serious morbidity and commonly causing outbreaks in Southeast Asia. For instance, neurocysticercosis is an infection caused by *Cysticercus cellulosae*, the metacestode larvae of *Taenia solium*, that can result in raised intracranial pressure, meningoencephalitis, focal neurological deficit and psychiatric symptoms [9]. Human trichinellosis has been reported in quite a number of countries around the world [10], with symptoms of malaise, anorexia, nausea, vomiting, abdominal pain, fever, diarrhea, and even death [11]. Paragonimiasis can lead to systemic infections with brain injuries that include hypertensive hemorrhage, coagulopathy, aneurysm, and arteriovenous malformation, sometimes even lead to death due to intracranial hemorrhage [12]. *Spirometra* spp. infections can frequently cause migrating granulomatous lesions in the subcutaneous tissue or pathological changes in the central nervous system [13]. Systemic and/or local eosinophilia is frequently observed in angiostrongyliasis. Several such outbreaks occurred recently in P.R. China and Thailand [14], [15]. Helminth infections, in particular, are associated with low socio-economic status and specific behavior (e.g., nutritional habits).

Given that multi-parasitic helminth infections are very common, we conducted the present study in order to establish a protein chip technique for the simultaneous detection of five major FBHs, namely cysticercosis, trichinellosis, paragonimiasis, sparganosis and angiostrongyliasis. There are three major reasons to do so. Firstly, similar symptoms presented in most of FBHs patients when they were admitted to the hospital could not simply be identified by conventional diagnostic approaches [16]–[29]. However, several kinds of parasites may be present in the same organs or the same symptoms may be caused by different parasites, which increase the difficulties of clinical differential diagnosis. For example, *Angiostrongylus cantonensis*, *C. cellulosae*, *Paragonimus westermani* and *Spirometra* spp., can parasitize human brains which cause dizziness, headache, aphasia, even epilepsy [12]–[15], [29], [30]. Low prevalence and minimal parasite burdens in some FBHs make it more difficult to detect the parasitic infection by conventional approach.

Secondly, population-based epidemiological studies require fast and high-throughput approach. Immunoassays for antibody and molecular detection have proven useful for epidemiological studies of FBHs for a long time [16]–[18], [21], [28], [29], [31], [32]. However, each of these methods and techniques has limitations in that they could not detect multiple parasitic infections by the application of just one test.

Thirdly, bio-chip technology has proven advantageous compared to traditional methods. It is a versatile, miniaturized, convenient approach that provides high-throughput diagnosis that can be adapted for a variety of screening uses [33]–[35]. A protein microarray is included in the bio-chip, which used to track the interactions and activities of proteins, and to determine their function, and determining function on a large scale [36]. There are a support surface such as a glass slide, nitrocellulose membrane, bead, or microtitre plate consisted of, to which an array of capture proteins is bound [37]. Probe molecules, typically labeled with a fluorescent dye, are added to the array. Reactions between the probe and the immobilized protein, which have been labeled with fluorescent dye, emit a fluorescent signal read by a laser scanner. These kinds of chips are rapid, automated, economical, and highly sensitive, consuming small quantities of samples and reagents [38].

Hence, the study is expected to produce a platform with higher sensitivity and specificity in population-based screening of multiple FBHs in comparison with traditional ELISA-based immunoassay.

## Materials and Methods

### Ethics statement

Ethical clearance for the retrospective use of human sera for test development and quality control was obtained from the Ethics Committee of the National Institute of Parasitic Diseases (NIPD), Chinese Center for Disease Control and Prevention (China CDC). The objectives, procedures and potential risks were orally explained to all participants. Collection of serum specimens was also conducted with approval from the Ethics Committee of NIPD, China CDC. All participants were given written informed consent to sign, and all child participants had a parent/guardian signing for them.

All animals were handled in strict accordance with good animal practice according to the Animal Ethics Procedures and Guidelines of the People's Republic of China, and the study was approved by the Laboratory Animal Welfare & Ethics Committee of NIPD, China CDC (Permit No: IPD2008-4).

### Human sera

Human sera were collected from both patients and healthy people. All of those positive sera harvested from patients who were confirmed either by “gold standard assays” that was pathological/parasitological examination or serum testing by immunological method (Table S1), or by combination of specific clinical symptoms and routine serological methods in accordance with the national criteria for clinical diagnosis of parasitic diseases. A total of 55 serum samples of patients infected with *C. cellulosae* were collected from Yunnan province, P.R. China. Among them, 21 patients had been confirmed microscopically by pathological anatomical diagnosis (PAD). The others were diagnosed by serological methods (using an ELISA kit produced by Combined Biotech Company, Shenzhen, Guangdong, P.R. China). All these patients presented with symptom of dizziness, raised intracranial pressure or emotional disturbance and a history of consuming raw pork. A total of 38 serum samples were obtained from patients with angiostrongyliasis. Among them, three patients were confirmed parasitologically (presence of larvae in the cerebrospinal fluid) and 35 patients were identified clinically along with a previous history of eating raw or undercooked food contaminated with parasites or intermediate hosts or transport hosts of *A. cantonensis*.. The diagnosis was arrived at Shenzhen Center for Diseases Control and Prevention, Shenzhen, P.R. China in accordance with the National Diagnosis Criteria of Angiostrongyliasis issued by Ministry of Health of P.R. China [39], in Shenzhen Center for Diseases Control and Prevention. In this group, eosinophilia (with at least 7% of the white cell count in the peripheral blood) and evidence of brain image abnormality had been observed. There was also high specificity in ELISA to the whole *A. cantonensis* antigen [14], [39], [40]. Furthermore, there were 45 serum samples from patients infected with *P. westermani* confirmed parasitologically, i.e. the eggs were detected in sputum or in the pleural fluid [25]. Another 42 serum samples of trichinellosis patients were collected from Yunnan province, P.R. China, including five cases confirmed pathologically or by finding *T. spiralis* larvae in muscle sections. The remaining 37 cases were diagnosed serologically by using an ELISA kit (Combined Biotech Company). Moreover, a total of 50 serum samples were collected from patients infected with *Spirometra* spp., in which 23 patients were confirmed by surgery (larvae of subcutaneous *Spirometra* spp. cysticerci)The others were diagnosed serologically by using an ELISA kit (Combined Biotech Company) (Table 1 and Table S1).

**Table 1:** Detection of food-borne helminthiases (FBHs) in various human sera using ELISA and protein microarrays with semi-purified antigens.

Serum samples	No. examined	No. positive of *C. cellulosae* sAg*		No. positive of *A. cantonensis* sAg*		No. positive of *P. westermani* sAg*		No. positive of *T. spiralis* sAg*		No. positive of *Spirometra* plerocercoids sAg*	
		ELISA	Arrays	ELISA	Arrays	ELISA	Arrays	ELISA	Arrays	ELISA	Arrays
Patients with *C. cellulosae*	55	47	49	0	0	0	0	0	0	2	3
Patients with *A. cantonensis*	38	0	0	35	35	0	0	0	0	0	0
Patients with *P. westermani*	45	0	0	0	0	39	40	0	0	4	5
Patients with *T. spiralis*	42	0	0	0	0	0	0	35	36	0	0
Patients with *Spirometra* plerocercoids	50	0	0	0	0	0	0	0	0	41	43
Patients with *T. gondii*	20	0	0	0	0	0	0	0	0	0	0
Patients with *C. sinensis*	20	0	0	0	0	3	3	0	0	0	1
Patients with *S. japonicum*	20	0	0	0	0	0	0	0	0	0	0
Patients with *A. lumbricoides*	20	0	0	0	0	0	0	0	1	0	0
Patients with *T. trichiura*	20	0	0	0	0	0	0	0	0	0	0
Patients with *A. duodenale*	15	0	0	0	0	0	0	0	0	0	0
Patients with *E. granulosus*	7	2	3	0	0	0	0	0	0	1	1
Patients with *T. saginata*	5	1	1	0	0	0	0	0	0	2	2
Patients with *T. asiatica*	4	1	1	0	0	0	0	0	0	0	0
Patients with *Filaria*	4	0	0	0	0	0	0	0	0	0	0
Healthy adult	80	0	0	0	0	0	1	0	0	0	0

* sAg: semi-purified antigens.

All serum samples obtained from Chinese patients infected with *Clonorchis sinensis*, *Schistosoma japonicum*, *Ascaris lumbricoides*, *Trichuris trichiura*, and *Ancylostoma duodenale* were diagnosed by parasitological examination, or the eggs were detected in feces [41]. A total of nine serum samples were collected from patients infected with *Taenia saginata* or *T. asiatica* in Guizhou province. Five patients infected with *T. saginata* and four infected with *T. asiatica* were diagnosed by identification of adult worms from their feaces after orally taken a concoction of areca and pumpkin seeds [42]. Seven patients infected with *Echinococcus granulosus* larvae were confirmed parasitologically after surgery, while the patients infected with *Toxoplasma gondii* and *Filaria* spp. were confirmed serologically by using ELISA kits (Combined Biotech Company). Sera of healthy adult people were collected from the serum bank stored at −80°C temperature at NIPD, China CDC and used as negative controls (Table 1 and Table S1). Information on the other patient groups including control groups can be found in the same place (Table S1).

### Parasites

Four parasite species are available in our laboratory, and one species was isolated from infected animals naturally. Thus, *A. cantonensis*, originally coming from an isolate collected from Fujian province, P.R. China, has been maintained in rats and the snail *Pomacea canaliculata* in our laboratory [43]. Rats were orally infected with the third-stage larvae (L3), and then adult worms were harvested from the lungs of the rat on day 33 post-infection [43]. Likewise, *P. westermani* adult worms were obtained from dog lungs 70 days post-infection with *P. westermani* metacercaria and metacercaria were collected from freshwater crab muscles in Fujian province, P.R. China [31]. *T. spiralis* infective larvae were collected directly from the skeletal muscle of naturally infected pigs in Henan province, P.R. China, by artificial digestion using pepsin according to standard procedures [44]. Plerocercoids of *Spirometra* spp., originally isolated from frogs in Guangdong province, P.R. China, were stored at −80°C in our laboratory.

### Antigens

Scolice antigens of *C. cellulosae* were kindly provided by Dr. R.L. Zhang of Shenzhen Center for Diseases Control and Prevention, Shenzhen, P.R. China. Whole worm antigens of *A. cantonensis*, *P. westermani*, *T. spiralis* and plerocercoids of *Spirometra* spp. were prepared following the standard protocol according to Ishida et al. [45] and Chen et al [43]. Briefly, parasites were washed in phosphate-buffered saline (PBS), pH7.4 3 times, then homogenized in PBS with a tissue homogenizer (Bio-Gen PRO200 Homogenizer, PRO Scientific Inc., Oxford, CT, USA) for 3 minutes. After tissue homogenization, all samples were sonicated at 13 Hg for 10 seconds and 25 cycles with an Ultrasonic Processor XL (Heat Systems Inc., Farmingdale, NY, USA). After keeping the sonicated homogenate at 4°C overnight, it was centrifuged at 10,000 rpm for 30 min at 4°C. The clear supernatant was gathered, the protein concentration measured by the Lowry method [46] and the liquid was stored at −80°C for further use.

### Electrophoresis

The crude protein preparations were separated according to molecular weight on 12% SDS-PAGE gel [47] and finally stained with 0.25% Coomassie blue G250 solution from Merck (http://www.merck.com).

### Immunoblot

Immunoblotting was performed as described previously with a slight modification [48]. The antigens of the different parasites were separated by electrophoresis on 12% SDS-PAGE, and blotted onto a nitrocellulose membrane (Bio-Rad, Hercules, CA, USA) using a semi-dry horizontal electro-transfer system (ATTO, Tokyo, Japan). After washing the membrane with PBS containing 0.05% tween-20 (PBST), blocking was done with 1% (v/v) bovine serum albumin (BSA) in PBST (BSA-PBST) at 4°C overnight. The strips were subsequently probed with sera from infected patients harboring parasites or normal sera for 1 h at room temperature (RT), and then incubated again for 1 h at RT with horseradish-peroxidase (HRP)-conjugated goat anti-human immunoglobulin G (IgG) (Sigma, St. Louis, MO, USA), diluted at 1/1000 in BSA-PBST. After incubation, the membrane was washed in PBST, followed by adding DAB+(3,3′-diaminobenzidine tetrahydrochloride) (Sigma) and the color bands monitored (Washed in PBST+DAB).

### Antigen purification

Semi-purification of antigens was performed following a protocol reported previously with some modifications [49]. Briefly, SDS-PAGE gel electrophoresis was performed to separate the various parasite antigens. After staining, the target bands were viewed and cut with a scalpel. Each band was broken into pieces of approximately 1×1 mm size and transferred into centrifuge tubes. PBS was subsequently added in sufficient quantity to cover the gel pieces. The gel pieces were centrifuged at 10, 000 rpm for 30 min at 4-°C and the homogenates kept at 4°C over night. The supernatants were gathered into dialysis membranes and subjected to dialysis in 2 liters of PBS at 4°C for 12 h with buffer solution changes and two repetitions of the procedure. The protein concentrations of the semi-purified antigens were measured by the Lowry method [46] after which the semi-purified antigens were placed in microcentrifuge tubes and stored at −80-°C until use.

### ELISA assays

Each well of polystyrene 96-well plates (BBI, Shanghai, P.R. China) were sensitized using a standardized quantity (1 µg) of semi-purified antigen, diluted in 100 µl 0.05 M bicarbonate buffer (pH 9.6) and then incubated overnight at 4-°C. After the incubation, the plates were manually washed three times with PBST after which the wells were blocked with BSA-PBST by incubating them at RT for 2 h. After removing the fluid, the different human sera (in 1∶100 dilution with BSA-PBST) were added to each well (100 µl/well) and incubated at 37°C for 1 h, followed by three washings with PBST. After this procedure, 100 µl of HRP-conjugated goat anti-human IgG (diluted 1/3000 in BSA-PBST) were put into the wells. The plates were incubated at 37°C for 45 min and washed three times in PBST after which the substrate solution (0.02% H_2_O_2_), 2 mg/ml o-phenylenediamine (www.Sigmaaldrich.com/) and 0.1 M citrate-phosphate buffer (pH 5.8) were added to each well (100 µl/well) followed by 5 min incubation at 37°C [43]. Finally, the reaction was stopped with 2 M H_2_SO_4_ (50 µl/well). The optical density (OD value) of the reaction product was read at 492 nm in an ELISA reader (Thermo Scientific, Waltham, MA, USA) [43]. The positive cutoff value was calculated as the mean OD value of the normal controls plus 2 standard deviations (SD).

### Protein microarray

The preparation of the protein microarray followed previous descriptions 38,50. Briefly, the semi-purified parasite proteins at a concentration of 0.2 mg/ml in spotting solution (9.3 mM phosphate buffer, pH 7.4) was spotted to each well of the microarrays (CapitalBio, Beijing, P.R. China) and incubated for 2 hrs at 37°C. The microarray contained an area spotted with cyanine dye 3 (Cy3)-labeled goat anti-human IgG (Sigma) as quality control, human IgG (Sigma-Aldrich) for a positive control and spotting solution only for a blank control.

A total of 365 human sera collected from patients infected with different parasites and 80 healthy individuals were examined by the protein microarray following previous descriptions [38], [50]. The microarray slides were blocked by adding BSA-PBST for 1 h at 37°C. After washing for 3×5 min with PBST, individual human sera were diluted 1/100 in BSA-PBST and then incubated for 1 h at 37°C. The slides were washed for 3×5 min in PBST and incubated for 1 h at 37°C with a 1∶1000 dilution of Cy3-labeled goat anti-human IgG. They were then washed for 3×5 min in PBST and scanned at 532 and 635 nm using a microarray scanner (CapitalBio). If the relative ratio of signal intensity (SI) was >2.0 (test relative to negative control >2.0), the response was considered as positive overall and the response statistically significant (*p*<0.05) compared to the negative control SI.

### Statistical analysis

All data were processed and analyzed with Microsoft Office Excel 2007 for Windows. The sensitivity, specificity, positive predictive value (PPV) and negative predictive value (NPV) were calculated relative to the results of “gold standard assays” described as above, or if parasitic eggs had been detected parasitologically in the feces from the patient from whom the serum came (Table S1). The 95% confidence intervals (CI) were determined as described previously [51], [52]. The Youden index, defined as sensitivity plus specificity minus 1, was used as a summary measure of the ability of a test to discriminate true positives from true negative results [53].

## Results

### Antigen purification and immunoblot

**Figure 1:**
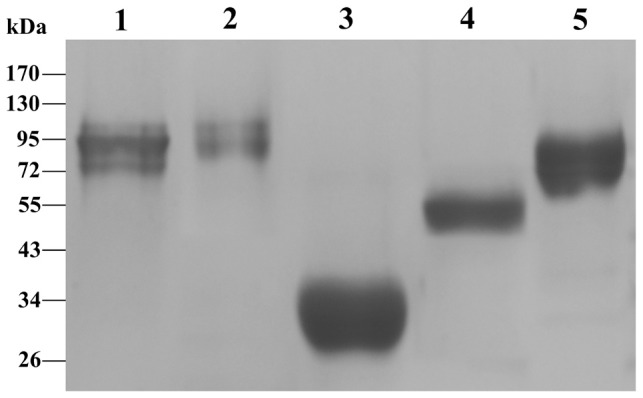
SDS-PAGE profile of semi-purified antigens from five parasites confirmed as food-borne helminthiases. Lanes 1 to 5 represent the semi-purified antigens with the molecular weight of 95, 98, 35, 55 and 90 kDa from *C. cellulosae*, *A. cantonensis*, *P. westermani*, *T. spiralis* and *Spirometra* plerocercoids, respectively.

After electrophoresis of the semi-purified antigens from each parasite investigated, immunoblotting with sera from patients infected with *C. cellulosae*, *A. cantonensis*, *P. westermani*, *T. spiralis* and *Spirometra* spp., respectively, was carried out to show the presence, place and relative purity of each antigen. The antigens were demonstrated as the 95 kDa band of *C. cellulosae*, the approximately 98 kDa band of *A. cantonensis*, the 35 kDa band of *P. westermani*, the 55 kDa band of *T. spiralis* and the 90 kDa band of *Spirometra* spp. plerocercoids (Figure 1).

**Figure 2:**
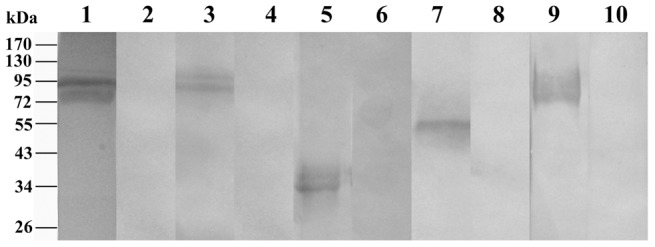
Immunoblot profiles of five parasites confirmed as food-borne helminthiases with corresponding sera. Lanes 1, 3, 5, 7 and 9 represent the semi-purified antigens of *C. cellulosae*, *A. cantonensis*, *P. westermani*, *T. spiralis* and *Spirometra* plerocercoids recognized by the corresponding sera of the patients, respectively. Lanes 2, 4, 6, 8 and 10 represent the semi-purified antigens of *C. cellulosae*, *A. cantonensis*, *P. westermani*, *T. spiralis* and *Spirometra* plerocercoids recognized by the sera of healthy adult, serving as an example.

Immunoblotting profiles obtained with sera from patients infected with *C. cellulosae*, *A. cantonensis*, *P. westermani*, *T. spiralis* and *Spirometra* spp., are shown in Figure 2. Each band represents a reaction with the semi-purified antigens of *C. cellulosae*, *A. cantonensis*, *P. westermani*, *T. spiralis* and *Spirometra* spp. Plerocercoids, respectively (Figure 2).

### ELISA assays

The outcome of the ELISA tests carried out with the 365 sera from patients infected with different parasites, as well as those from the 80 healthy individuals, are summarized in Table 1.

The specificities and sensitivities of the ELISA tests ranged from 97.7% (95% CI: 96.2–99.2%) to 100.0%, and from 82.0% (95% CI: 71.4–92.6%) to 92.1% (95% CI: 83.5–100.0%), respectively. The Youden index varied from 0.80 to 0.92 (Table 2).

**Table 2:** Sensitivities, specificities, positive predictive values (PPVs), negative predictive values (NPVs) and Youden indices of ELISA and protein microarrays.

	*C. cellulosae* sAg*		*A. cantonensis* sAg*		*P. westermani* sAg*		*T. spiralis* sAg*		*Spirometra* plerocercoids sAg*	
	ELISA	Arrays	ELISA	Arrays	ELISA	Arrays	ELISA	Arrays	ELISA	Arrays
No. of true positives	47	49	35	35	39	40	35	36	41	43
No. of false positives	4	5	0	0	3	4	0	1	9	12
No. of true negatives	386	385	407	407	397	396	403	402	386	383
No. of false negatives	8	6	3	3	6	5	7	6	9	7
Sensitivity (%) [95% CI**]	85.5 [76.2–94.8]	89.1 [80.9–97.3]	92.1 [83.5–100.0]	92.1 [83.5–100.0]	86.7 [76.8–96.6]	88.9 [79.7–98.1]	83.3 [72.0–96.6]	85.7 [75.1–96.3]	82.0 [71.4–92.6]	86.0 [76.4–95.6]
Specificity (%) [95% CI**]	99.0 [98.0–100.0]	98.7 [97.6–99.8]	100.0	100.0	99.3 [98.5–100.0]	99 [98.0–100.0]	100.0	99.8 [98.4–100.0]	97.7 [96.2–99.2]	97.0 [95.3–98.7]
PPV (%) [95% CI**]	92.2 [84.8–96.6]	90.7 [83.0–98.4]	100.0	100.0	92.9 [85.1–100.0]	90.9 [82.4–99.4]	100.0	97.3 [92.1–100.0]	82 [71.4–92.6]	78.2 [67.3–89.1]
NPV (%) [95% CI**]	98.0 [96.6–99.4]	98.5 [97.3–99.7]	99.3 [98.5–100.0]	99.3 [98.5–100.0]	98.5 [97.3–99.7]	98.8 [97.7–99.9]	98.3 [97.0–99.6]	98.5 [97.3–99.7]	97.7 [96.2–99.2]	98.2 [96.9–99.5]
Youden's index	0.85	0.88	0.92	0.92	0.86	0.88	0.83	0.86	0.80	0.83

* sAg: semi-purified antigens;

** CI: confidence interva.

### Protein microarray

**Figure 3:**
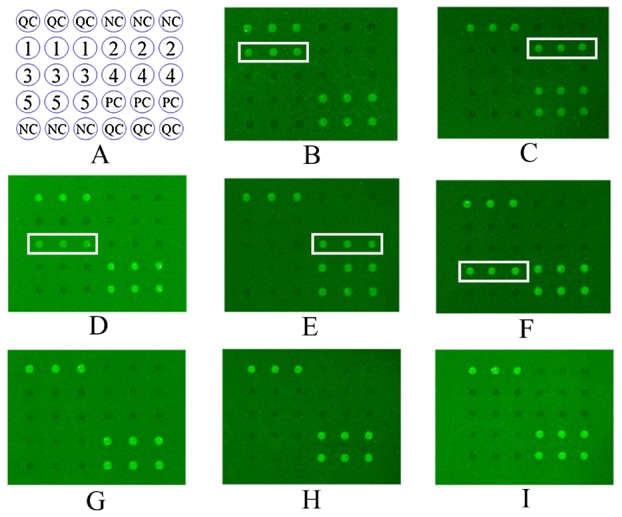
Protein microarrays probed with human sera with various food-borne helminthiases. Disposition of microarray (A), the semi-purified antigens of the parasites recognized by the sera from patients infected with *C. cellulosae* (B), *A. cantonensis* (C), *P. westermani* (D), *T. spiralis* (E), *Spirometra* plerocercoids (F), healthy adult (G), *A. lumbricoides* (H) and *S. japonicum* (I) with the method of protein microarrays, respectively. On panel A, QC = quality control; PC = positive control of arrays; NC = negative control of arrays; Spot 1 to 5 = the semi-purified antigens with the molecular weight of 95, 98, 35, 55 and 90 kDa from *C. cellulosae*, *A. cantonensis*, *P. westermani*, *T. spiralis* and *Spirometra* plerocercoids, respectively.

The results of the 365 sera from patients and the 80 sera from the healthy individuals examined by protein microarray using the semi-purified antigens are summarized in Table 1 and also shown in Figure 3.

The specificities and sensitivities of protein microarray ranged from 97.0% (95% confidence interval (CI): 95.3–98.7%) to 100.0%, and from 85.7% (95% CI: 75.1–96.3%) to 92.1% (95% CI: 83.5–100.0%), respectively. The Youden index of protein microarray varied from 0.83 to 0.92 (Table 2). The results show that, under the condition of using the same antigen, most of the Youden indices of protein microarray results were higher than those obtained from the ELISA tests used for single parasite detection.

## Discussion

Recently, considerable efforts have been dedicated to establishing useful and convenient methods for sero-diagnosis of FBHs [54]–[57]. However, the procedures are commonly limited as they are time-consuming, require intensive labor, or produce low specificity. With the development of etiological detection technology, microarrays have rapidly expanded into the area of biological research, including gene expression, signal transection, genome mismatch scanning, and protein trafficking. Among the many types of microarrays, the protein microarray offers an opportunity to study the full spectrum of protein attributes in a parallel, miniaturized, and automated fashion representing a significant shift from the traditional “one protein at a time” methods [58], [59]. Furthermore, the method not only aids in improved diagnosis, but also identifies autoantibody signatures that may represent disease subgroups, early diagnostics [60], [61] and facilitates the analysis of the outcome of vaccine trials [62].

In this study, a protein chip was used as a diagnostic tool for FBHs to detect disease-specific antibodies. Our study results indicate that different parasites have variant diagnostic antigens, therefore they can be used for specific and differential detections. For example, the proteins of *T. solium* cysticerci or *C. cellulosae* with molecular weights of 100, 95 or 26 kDa have been used in confirmatory tests for neurocysticercosis-positive cases [49]. Meanwhile, the 98 kDa antigen of *A. cantonensis* as a candidate antigen can be used for the diagnosis of angiostrongyliasis [43]. The 35 kDa protein spotted into protein chip could be used for the diagnosis of paragonimiasis, although further verification is needed to investigate if the protein is the same as the 35 kDa in *P. westermani* adult worm soluble antigens [63]. Moreover, it has been reported that the specificities using well-archived serum samples of sera-diagnosis for detection of different FBHs are ideal with positive percentages ranging from 83% to 100%, while the sensitivities ranged from 50 to 98% [16]–[18], [21], [28], [29], [64], [65]. In spite of the semi-purified proteins prepared for each parasites in the study, such as the 95 kDa protein of *C. cellulosae*, the 98 kDa protein of *A. cantonensis*, the 35 kDa protein of *P. westermani*, the 55 kDa protein of *T. spiralis* and the 90 kDa protein of *Spirometra* spp. plerocercoids, our results show that the approach of protein microarray display higher specificity in the detection of each infection, ranging from 97.0% to 100.0%.

It was also displayed that the protein microarray had excellent NPV (98.2%–99.3%) and PPV (78.2%–100.0%). In contrast, both of the values varied slightly in ELISA. These data indicate that there is a high probability that the protein microarray negatives are truly uninfected and that the positive results with the protein microarray indeed infected in the population investigated. When it comes to using the same antigen, most of the Youden indices for the protein microarray were a little higher than those produced by the ELISA test used for individual FBH detection. In terms of the advantage of this technology, the protein microarray can be used for epidemiological screening of FBHs on a large scale and play a role in prevention and treatment of parasitic diseases.

Protein microarray technology has played a significant role in the study of protein-protein interactions and been used for the identification of antigenic targets of serum autoantibodies in a variety of diseases. More recently, this technology has been applied in the detection and the identification of autoantibody signatures in five FBHs. The development of 3-dimensional surfaces will support a high-throughput approach which can detect more than one pathogen with only one chip and avoid time-consuming, high costs, and labor required for conventional tests, such as ELISA and colloidal gold testing. The two indices, e.g. predictive values influenced by prevalence of the detected population, and properties of diagnostics including sensitivity and specificity, are the main indices to assess the yield of screening test when it is used in the field [51]. This research should be seen as a primary attempt in our laboratory, since this method of protein microarray did not generate significant differences with respect to sensitivity. Hence, it is expected that the application of monoclonal antibody might increase the sensitivity of the method in the future. Moreover, it is useful that this chip can be used for clinical detections as well as field screening. In our further study, we aim to produce monoclonal antibodies with specificities for each of the five helminthiases for protein chip testing.

In conclusion, the present study established a protein microarray for the rapid, sensitive and high-throughput diagnosis of cysticercosis, trichinellosis, paragonimiasis, sparganosis and angiostrongyliasis. This technique may contribute to better control and prevention of FBHs.

## Supporting Information

Table S1 **The diagnoses of 365 patients by parasitological or serological methods. Found at: doi:10.1371/journal.pntd.0000771.s001 (0.14 KB DOC).**
(DOCX)
