# Supplementary material for: A Protein Microarray for the Rapid Screening of Patients Suspected of Infection with Various Food-Borne Helminthiases
Source: PLoS Negl Trop Dis. 2012 Nov 29;6(11):e1899. doi: 10.1371/journal.pntd.0001899 (PMC3510079; doi:10.1371/journal.pntd.0001899)
Supplement: Table S1 — The diagnoses of 365 patients by parasitological or serological methods. Found at: doi:10.1371/journal.pntd.0000771.s001 (0.14 KB DOC). (DOCX) [file pntd.0001899.s001.docx]

**Table S1**

**The diagnoses of 365 patients by the gold standard assay or clinical diagnosis.**

| **Patients** | **Gold standard assay** | **No. of patients diagnosed by gold standard assay** | **No. of patients diagnosed by clinical diagnosis combination of clinical symptoms and serological tests** | **Total** |
| --- | --- | --- | --- | --- |
| Patients with *C. cellulosae* | Pathological examination (*C. cellulosae* in pathological section) | 21 | 34 | 55 |
| Patients with *A. cantonensis* | Parasitological examination (larvae in cerebrospinal fluid) | 3 | 35 | 38 |
| Patients with *P. westermani* | Sputum smear examination (Eggs in sputum or pleural fluid) | 45 | 0 | 45 |
| Patients with *T. spiralis* | Pathological examination (*T*. *spiralis* larvae in muscle) | 5 | 37 | 42 |
| Patients with *Spirometra* plerocercoids | Pathological examination (Surgery operation to find the larvae of subcutaneous cysticerci of *Spirometra* spp.) | 23 | 27 | 50 |
| Patients with *T. gondii* | Pathological examination　(*T*. *gondii* cysts or tachyzoite in body fluid) | 0 | 20 | 20 |
| Patients with *C. sinensis* | Fecal examination (Eggs in feces) | 20 | 0 | 20 |
| Patients with *S. japonicum* | Fecal examination (Eggs in feces) | 20 | 0 | 20 |
| Patients with *A. lumbricoides* | Fecal examination (Eggs in feces) | 20 | 0 | 20 |
| Patients with *T. trichiura* | Fecal examination (Eggs in feces) | 20 | 0 | 20 |
| Patients with *A. duodenale* | Fecal examination (Eggs in feces) | 15 | 0 | 15 |
| Patients with *E. granulosus* | Pathological examination (Symptom of hepatic cysticerci of *E. granulosus* confirmed by operation to find the larvae in organs) | 7 | 0 | 7 |
| Patients with *T. saginatus* | Fecal examination (The adult worms found after expelling by decoction of areca and pumpkin seeds) | 5 | 0 | 5 |
| Patients with *T. asiatica* | Fecal examination (The adult worms found after expelling by decoction of areca and pumpkin seeds) | 4 | 0 | 4 |
| Patients with *Filaria* | Blood smear examination (Microfilariae in peripheral blood) | 0 | 4 | 4 |
